# Spatial distribution and incidence trend of human alveolar echinococcosis in southwest Germany: increased incidence and urbanization of the disease?

**DOI:** 10.1007/s15010-020-01479-4

**Published:** 2020-07-16

**Authors:** Matthias C. Mueller, Michael Marx, Gabriele Peyerl-Hoffmann, Winfried V. Kern

**Affiliations:** 1 Division of Infectious Diseases, Department of Medicine II, Medical Center, Faculty of Medicine, University of Freiburg, Hugstetter Straße 55, 79106 Freiburg, Germany; 2 Department of Infection Medicine, Medical Care Center, MVZ Clotten, Freiburg, Germany

**Keywords:** *Echinococcus multilocularis*, Alveolar echinococcosis, Urban, Germany

## Abstract

Ecology and epidemiology of *Echinococcus multilocularis* and human alveolar echinococcosis (AE) are changing in Central Europe. Our data from a regional referral center for AE in southwest Germany suggest rising regional incidence for AE (annual incidence per 100,000 population 2004–2011: 0.12; 2012–2019: 0.20) and emerging urban AE (of 7 cases of AE in Freiburg city dwellers none was diagnosed before 2012) calling for an intensification of *E. multilocularis* and AE surveillance and of AE prevention measures.

**Electronic supplementary material:**

The online version of this article (10.1007/s15010-020-01479-4) contains supplementary material, which is available to authorized users.

## Introduction

Alveolar echinococcosis (AE) is a life threatening helminth disease caused by larval (metacestode) stages of the fox tapeworm, *Echinococcus multilocularis.* Human AE is acquired by peroral infection with eggs released into the environment with the feces by the definitive host which includes in Europe mainly foxes and to a much lesser extend domestic dogs and cats. Rodents are intermediate hosts. After ingestion of eggs and penetration of the wall of the small intestine, metacestodes show an infiltrative growth pattern primarily in the liver but virtually all organs can be involved. The disease becomes clinically apparent after 5 to 15 years of incubation period. Untreated AE has a very poor outcome, and only early stages of disease or restricted focal appearance may qualify for curative surgical treatment. Introduction of long-term parasitostatic treatment with benzimidazoles improved survival of inoperable AE patients dramatically.

*Echinococcus multilocularis* and AE occur in many countries of the northern hemisphere. In Europe, the epidemiology of *E. multilocularis* and AE is changing since the 1990s. AE has been known to be endemic in the Central European AE belt including eastern France, southern Germany, northern Switzerland, and western Austria since the second half of the 19th century. In the last decades, the parasite and the disease have spread to northern Germany and most of mainland Europe with the exception of southern European and most Scandinavian countries. In addition to the geographically spread, the biomass of the parasite has grown drastically in the epidemic regions due to sharply increasing fox populations and parasites prevalence rates [1, 2]. Moreover, foxes, for most of the 20th century not known to occur in larger towns and cities in continental Europe, established large self-sustaining populations within villages and towns since the middle of the 1990s with higher fox population densities than in rural settings and high prevalence of *E. multilocularis* [3]. Surveys in the late 90s in major European cities such as Stuttgart, Geneva, and Zurich in the Central European AE belt revealed parasite prevalence rates of 17%, 43%, and 44%, respectively [3, 4]. Elevated parasite biomass in densely populated urban areas results in a rising infection pressure and may lead to an increased incidence of human AE. Indeed, data from the 2000s show increasing incidence rates in Switzerland, France, Austria and Germany compared to the 1990s [5–7] and in Switzerland human AE is increasingly reported from resident of urban areas but cases of human AE in urban dwellers in Germany remain rare [3, 8]. Due to this evolution, *E. multilocularis* is considered a serious public health threat in Europe [9].

In Germany, AE is a rare disease with 165 reported cases in the years 2012 to 2016, of those 80% were occurring in the epidemiological hot spots of the federal states of Baden-Württemberg and Bavaria [7]. Since 2001, AE is a notifiable disease but reporting is estimated to be poor, failing to detect 67% of AE cases in Germany [7, 10]. The early identification of emerging hotspots of human AE incidence is crucial for the implementation of preventive measures. Here we report the epidemiological data of human AE cases in treatment at a regional referral center at the Medical Center of the University of Freiburg situated in the epicenter of European AE epidemic area in the southwest of Germany.

## Methods

### Ethics consideration

The study was approved by the institutional ethics review board of the medical center of the University of Freiburg (No.: 363/18). All patients eligible for this study were tried to be contacted for provision of informed consent. No patient denied to participate in this study.

### Study setting/design

This study has a retrospective study design. AE was defined following the case definition of WHO Informal Working Group on Echinococcosis (WHO-IWGE) [11]. All patients who had presented with probable or confirmed diagnosis of AE according to WHO-IWGE definition at the Medical Center of the University of Freiburg between January 2004 and November 2019 were included in the study. Data were extracted from the patient’s records. AE cases have been reported to the Echinococcus registry of the University of Ulm in the years 2003 to 2013 [7]. The Medical Center of the University of Freiburg has a catchment area that covers the middle and southern part of the Black Forest mountain range and the Upper and Higher Rhine Valley up to the Lake of Constance with a population of about 2.3 Million (local government unit “Regierungsbezirk” Freiburg) and is the only referral center for AE in the region. The city of Freiburg with a population of about 220,000 is bordered by the forested hills of the Black Forest and located in the center of the Central European AE belt in the southwest of the federal state of Baden-Württemberg in Germany.

## Results

A total of 58 patients with *E. multilocularis* infection were enrolled in the study. Table 1 summarizes the baseline characteristics. Overall, 48.3% of participants were female. At time of diagnosis of *E. multilocularis* infection, patients were in a median 63.0 years old (ranges: 12.9–86.0) and 43.1% of them had an immunosuppressive condition including 5 patients (8.5%) with solid organ transplant without variation in the observed time periods.

**Table 1:** Baseline characteristics of patients with alveolar echinococcosis treated at the Medical Center of the University of Freiburg between January 2004 and November 2019

Observation period	Total	2004–2011	2012–2019
N	58	22	36
Female *n* (%)	28 (48.3)	12 (54.6)	16 (44.4)
Age^a^ (years) [median (range)]	63.0 [12.9–86.0]	61.4 [12.9–79.4]	64.5 [19.9–86.0]
Place of residence city of Freiburg^a^	7 (12.1)	0	7 (19.4)
Immunosuppression^a^	26 (43.1)	9 (42.9)	17 (47.2)
CID	9 (15.5)	1 (4.8)	8 (22.2)
Diabetes mellitus	6 (10.5)	3 (14.3)	3 (8.3)
Solid tumor	5 (8.7)	2 (9.5)	3 (8.3)
Kidney transplantation	4 (6.9)	3 (14.3)	1 (2.7)
Lung transplantation	1 (1.7)	0	1 (2.7)
Liver cirrhosis	1 (1.7)	0	1 (2.7)
Therapy			
Curative surgery	31 (53.5)	15 (68.2)	16 (44.4)
Exclusively parasitostatic therapy	22 (38.0)	6 (27.3)	16 (44.4)
Watch and wait	5 (8.6)	1 (4.6)	4 (11.1)
Death related to AE	1	0	1

*AE* alveolar echinococcosis, *CID* chronic inflammatory disease including sarcoidosis, chronic inflammatory bowel disease, rheumatoid arthritis, giant-cell arteritis

^a^At the moment of diagnosis

**Fig. 1:**
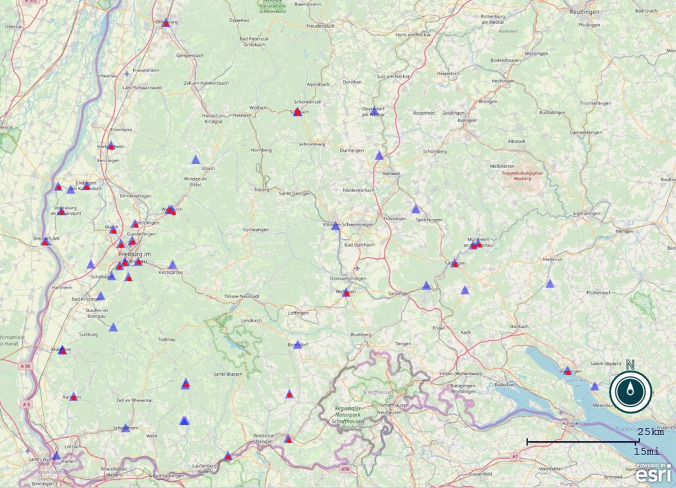
Places of residence of patients at the time of diagnosis of alveolar echinococcosis treated at the regional referral center at the Medical Center of the University of Freiburg between January 2004 and November 2019. Red dots: diagnosis between the years 2012 and 2019

AE incidence in the years 2012 to 2019 increased by 63.6% compared to the years 2004 to 2011. Between 2004 and 2011 a mean of 3.69 patients per year were diagnosed with AE. Since 2012 numbers of patients with initial diagnosis of AE exceed the yearly mean considerably in 5 years. On a population level average annual incidence per 100,000 population increased from 0.12 between 2004 and 2011 to 0.20 between 2012 and 2019. Seven patients living in the city of Freiburg were diagnosed with AE, none of them in the years before 2012 (Fig. 1). All seven patients lived all their live or at least 25 years in the city of Freiburg. Five patients lived in the urban area of the town, two patients lived in suburban areas, and one of them was a farmer. None of the other 6 patients had classic risk factors for AE like owning a dog or vegetable garden.

## Discussion

Our study shows increasing numbers of human AE in the endemic area in southwest Germany since 2011 and in Freiburg city dwellers which was an unknown phenomenon until 2012. Increasing numbers of AE seem not be due to an increasing number of susceptible persons, as proportions of patients with immunosuppressive state remain stable over the time of observation.

**Fig. 2:**
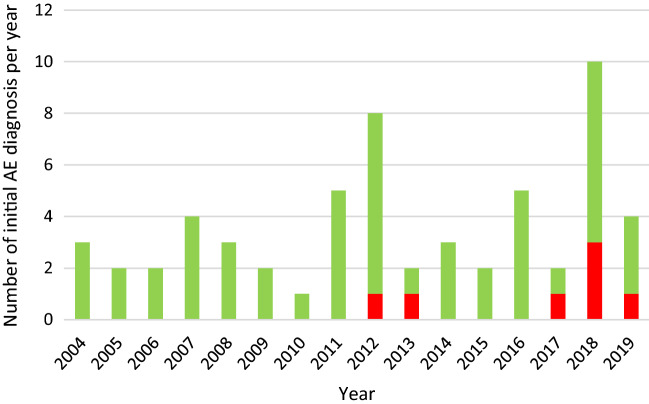
Numbers of human alveolar echinococcosis per year of diagnosis treated at the regional referral center at the Medical Center of the University of Freiburg between January 2004 and November 2019. Red bars: patients with residence in the city of Freiburg

In line with our results, there is clear evidence of increasing incidences of human AE in the neighboring countries Switzerland and France, as well as Austria since the beginning of the 20th century [6, 9, 12]. In Switzerland for example, a statistically significant twofold increase of the average annual incidence was reported from 0.10 during 1993–2000 to 0.26 during 2001–2005 per 100,000 population [6]. In Germany, numbers of registered AE cases are rising since the 90s, most probably to higher incidence of human AE but data is less robust as AE is a notifiable disease only since 2001 [7]. Increasing AE incidence in the Central European AE belt is believed to be founded in the steeply rising parasite biomass due to a sharply increasing fox population and parasite prevalence since introduction of oral vaccination campaigns against rabies in foxes in the early 80ies [13]. In southern Germany fast-growing fox population showed increasing parasite prevalence to up to 75% and 80% in 1995 and 2002/2003 in the most affected areas resulting in tenfold higher biomass estimates in the period 1995–2000 compared to estimates before 1990 [1, 2, 14, 15] (Fig. 2).

Simultaneously, foxes colonized urban areas. Foxes living in urban areas were a known phenomenon in Great Britain due to high fox population densities in the absence of livestock reducing rabies. In Germany foxes were regularly sighted in urban areas in the middle of the 90s and established resident self-sustained fox populations in German and other European cities in the 2000s with high population densities and *E. multilocularis* prevalence rates of up to 44% [4]. In contrast to the rising density of fox populations from rural through peri-urban to urban areas, *E. multilocularis* frequencies show the opposite trend probably due a decreasing availability of the intermediate hosts in increasingly urbanized areas [16]. Against this background, peri-urban areas, parks, urban gardens and wasteland with high population densities of foxes, intermediate hosts and humans might be the hotspot of *E. multilocularis* transmission. In a study in the outskirts of Zurich for example, up to 50% of 57 fox droppings were tested positive for *E. multilocularis* during winter with an important seasonality effect [16]. With the establishment of urban wildlife cycles of *E. multilocularis* dogs may be become an important source of human infection. Prevalence of *E. multilocularis* in dogs generally is much lower than in foxes (approximately 0.3–0.4%), but may be elevated in dogs with regular access to infected rodents. In an epidemic area in rural western Switzerland 7% of 86 dogs were identified as carriers of *E. multilocularis* [17]. Lower parasite prevalence may be outweighed by high dog population density in urban areas; it has been calculated that dogs may contribute to 7–19% of environmental contamination with *E. multilocularis* eggs under urban conditions [18]. Frequent and close contacts to humans further increases risk of transmission. In China where dogs became involved in the parasite’s life cycle, prevalence of human AE can reach up to 4% [3].

In light of the above, emerging cases of AE in residents of the densely populated outskirts and urban center of Freiburg give rise to concern. There is no established surveillance system or data on fox population and *E. multilocularis* biomass density in the region of Freiburg. But in parallel to supraregional and international trends, density of urban fox population in Freiburg drastically increased in the last decades suggesting an associated augmentation of *E. multilocularis* biomass especially in the gardens, local recreation areas, and outskirts of the city. Taking into account the incubation period of 5–15 years, the now observed cases of urban human AE may be a result of the increased infection pressure in the last decades and the first signal of elevated AE incidence.

Our survey shows more AE cases in the Freiburg region and the Upper Rhine Valley than previous epidemiological studies covering the years 1992 to 2016, which is probably due the recent emergence of AE in this region and delayed reporting [7].

Due to the study design, the small number of cases and the impossibility to determine the actual geographical location of *E. multilocularis* transmission, our data are not able to document a statistically significant increasing incidence and urbanization of human AE. But against the background of the changes in the ecology and epidemiology of *E. multilocularis*, the long incubation time and the devastating nature of human AE, we belief that the here presented observations should prompt measures to assess the extent of *E. multilocularis*-related public health threat in the densely populated region of Freiburg as guidance for future prevention strategies.

## Electronic supplementary material

Below is the link to the electronic supplementary material.

Supplementary material 1 (XLS 39 kb)

## Data Availability

Research datasets is submitted as electronic supplementary material.
